# A Nomogram for Predicting Cancer-Specific Survival in Children With Wilms Tumor: A Study Based on SEER Database and External Validation in China

**DOI:** 10.3389/fpubh.2022.829840

**Published:** 2022-04-07

**Authors:** Xiaojun Tan, Jinkui Wang, Jie Tang, Xiaomao Tian, Liming Jin, Mujie Li, Zhaoxia Zhang, Dawei He

**Affiliations:** ^1^Department of Urology, Children's Hospital of Chongqing Medical University, Chongqing, China; ^2^Department of Urology, Nanchong Central Hospital, The Second Clinical Medical College, North Sichuan Medical University, Nanchong, China; ^3^Chongqing Key Laboratory of Children Urogenital Development and Tissue Engineering, Chongqing, China; ^4^Chongqing Key Laboratory of Pediatrics, Chongqing, China; ^5^Ministry of Education Key Laboratory of Child Development and Disorders, Chongqing, China; ^6^National Clinical Research Center for Child Health and Disorders, Chongqing, China; ^7^China International Science and Technology Cooperation Base of Child Development and Critical Disorders, Chongqing, China; ^8^Department of Epidemiology, Public Health School, Shenyang Medical College, Shenyang, China

**Keywords:** nomogram, cancer-specific survival, Wilms tumor, children, SEER

## Abstract

**Background:**

Wilms tumor (WT) is the most common tumor in children. We aim to construct a nomogram to predict the cancer-specific survival (CSS) of WT in children and externally validate in China.

**Methods:**

We downloaded the clinicopathological data of children with WT from 2004 to 2018 in the SEER database. At the same time, we used the clinicopathological data collected previously for all children with WT between 2013 and 2018 at Children's Hospital of Chongqing Medical University (Chongqing, China). We analyzed the difference in survival between the patients in the SEER database and our hospital. Cox regression analysis was used to screen for significant risk factors. Based on these factors, a nomogram was constructed to predict the CSS of children with WT. Calibration curve, concordance index (C-index), the area under the receiver operating curve (AUC) and decision curve analysis (DCA) was used to evaluate the accuracy and reliability of the model.

**Results:**

We included 1,045 children with WT in the SEER database. At the same time, we collected 112 children with WT in our hospital. The Kaplan-Meier curve suggested that children in China with WT had a higher mortality rate than those in the United States. Cox regression analysis revealed that age, lymph node density (LND), and tumor stage were significant prognostic factors for the patients in the SEER database. However, the patients in our hospital only confirmed that the tumor stage and the number of positive regional lymph nodes were significant factors. The prediction model established by the SEER database had been validated internally and externally to prove that it had good accuracy and reliability.

**Conclusion:**

We have constructed a survival prognosis prediction model for children with WT, which has been validated internally and externally to prove accuracy and reliability.

## Introduction

Wilms tumor (WT) is derived from embryonic kidney tissue and is the most common kidney tumor in children, accounting for about 90% of kidney tumors (1). At present, in high-income countries, with modern multidisciplinary comprehensive treatment, the survival rate of children with WT can reach more than 90% (2–4). The International Society of Pediatric Oncology Renal Tumor Study Group (SIOP-RTSG) and Children's Oncology Group Renal Tumor Committee (COG-RTC) of the United States and Canada have been committed to improving the survival rate of tumors in children (5, 6). The difference is that COG-RTC advocates surgical resection first, while SIOP-RTSG advocates chemotherapy first. However, in underdeveloped countries, the late diagnosis of tumors in children and the higher recurrence rate are important factors for high child mortality (7). In China, the incidence of WT in children is about 330 per million (8). However, the incidence of WT in children in the United States is 7-10 per million (9). Therefore, the morbidity and mortality of WT in children in China are particularly cruel.

A previous study had developed and validated a nomogram for predicting the CSS of patients with WT, showing that age, tumor size, and the number of lymph nodes (LNs) examined were essential factors for the prognosis (10). In addition, some studies have also proved that age, tumor size, and tumor stage were factors influencing the forecast of WT (11, 12). Lymph node density (LND) has been proven to have significant prognostic value in colon cancer and rectal cancer (13). A study showed that the overall survival rate of children with WT was closely related to LND (14).

AI is already widely used in health care. Awais et al. (15) used texture analysis to classify the abnormal areas caused by oral cancer. Ghayvat et al. (16) apply blockchain to the management of medical big data. Mishra et al. (17) applied intelligence drive to multilevel assessment of psychological disorders. The nomogram is a numerical estimation of various clinicopathological factors to predict the occurrence of an outcome event. At present, the nomogram has been widely used to indicate the survival and prognosis of multiple cancers (18, 19). Although Tang et al. (20) have constructed a nomogram for CSS prediction in patients with WT, there is no study have been conducted for external validation.

Our purpose is to compare children's mortality and prognostic factors with WT between the United States and China. Then, the cases in the SEER database were used to construct a predictive prediction model for children with WT, and the patients from a children's hospital in China were used for external validation.

## Materials and Methods

### Data Source and Data Extraction

We collected clinicopathological data of all children under 18 with WT from the Surveillance, Epidemiology, and End Results (SEER) database. The SEER database is a cancer database in the United States, including 18 cancer registries and covering 28% of the US population (21). At the same time, we collected the clinicopathological information of all children with WT in Children's Hospital of Chongqing Medical University(CHCMU, Chongqing, China) from 2013 to 2018. The Ethics Committee approved our study of Children's Hospital of Chongqing Medical University, and the patient's guardian provided written informed consent.

**Figure 1:**
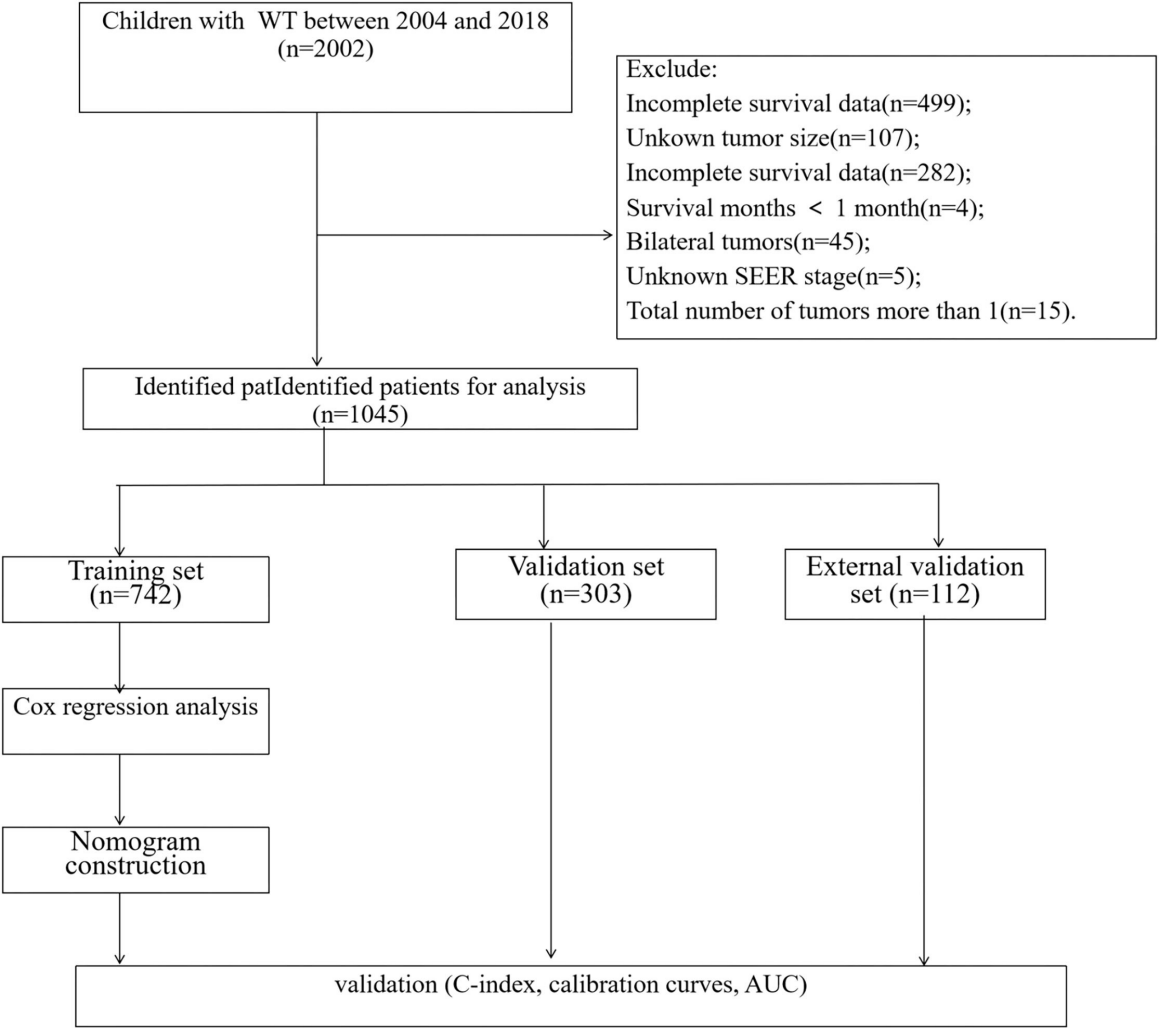
The flowchart of including and dividing patients.

Patients' clinicopathological data include age, sex, laterality, tumor stage, tumor size, number of lymph nodes (LNs) examined, number of positive lymph nodes, LND, surgery, and chemotherapy. The selection criteria are (1) Age ≤18 years; (2) International Classification of Oncological Diseases (ICD-O-3), code 8960; (3) Unilateral renal cancer. The exclusion criteria are (1) incomplete survival data; (2) the tumor size was unknown; (3) LNs examined and positive LNs were unknown; (4) Bilateral tumors; (5) the survival time was <1 month; (6) Unknown SEER stage. The patient screening process in the SEER database is shown in Figure 1.

LND is the number of positive regional LNs divided by the number of regional LNs examined. Age, tumor size, number of positive LNs, number of LNs examined, LND were continuous variables, expressed in median and inter-quartile ranges. The SEER stage was divided into three stages: local, regional and distant.

### Follow-Up of Patients

We followed up the children with WT in our hospital through outpatient clinic, hospitalization, telephone and letter. The start date of the follow-up was the first day after surgery and ended on March 1, 2019. The follow-up index was cancer-specific survival time, and the outcome event was death.

### Univariate and Multivariate Cox Regression Analysis

We randomly divided the 1045 children collected in the SEER database into a training set of 70% (*n* = 742) and a validation set of 30% (*n* = 303). Univariate and multivariate Cox proportional hazard risk models were used to evaluate independent risk factors for children with WT, and the hazard ratio (H.R.) and 95% confidence interval (CI) were recorded simultaneously.

### Nomogram Construction for CSS

The independent risk factors identified by cox regression analysis were used to construct a nomogram. When constructing the multivariate Cox regression, we obtained the regression coefficient β (coef) for each variable; Nomogram would normalize the regression coefficients and display them as risk scores on a number line.

### Nomogram Validation

The concordance index (C-index), the area under the receiver operating characteristics curve (AUC), and calibration curve were used to evaluate the accuracy and discrimination ability of the nomogram. Decision curve analysis (DCA) is a method for assessing the value of a model based on the net benefit under the estimated risk threshold (22). We used DCA to evaluate the clinical application value of the nomogram.

### External Validation and Clinical Utility

C-index, AUC and DCA were used for external validation to identify the accuracy and discrimination ability of the nomogram. In addition, we divided all patients into high-risk and low-risk groups according to their risk values on the nomogram. We used the Kaplan-Meier curve and log-rank test to test the difference in survival between the two groups.

### Statistical Analysis

For children in our hospital, the Kaplan-Meier method was used to estimate CSS. CSS was defined as the death of patients due to cancer, including metastasis, recurrence, or any related factors. Continuous variables were represented by median and inter-quartile range, and many-Whitney *U*-test was used to compare differences between groups. The comparison of categorical variables used the Pearson X2 test. All statistical analyses used R software (version 3.4.1; http://www.Rproject.org) and SPSS 24.0 (IBM Corp, Armonk, New York, USA). A *P*-value < 0.05 was considered statistically significant.

## Results

### Clinical Features

We included a total of 1,045 children with WT from the SEER database. The clinicopathological characteristics of the training set and the validation set are shown in Table 1. The median age of the patient was 3 years old (quartile, 1–5 years old). The median size of the children's tumor was 110 mm (quartile, 83–135 mm). The median number of regional LNs examined in the patient was 5 (quartile, 3 to 9). Among them, there were fewer males (47.46%), most patients had undergone chemotherapy (91.39%), lymph node-positive patients accounted for only 19.6%, and SEER stage was as follows: local accounted for 41.63%, regional accounted for 36.27%, and distant accounted for 22.11%. There was no significant difference between the patients in the training set and the validation set.

**Table 1:** Clinicopathological characteristics of children with WT in SEER database.

	**Total**	**Training set**	**Validation set**	
	***N* = 1045**	***N* = 742**	***N* = 303**	***p***
Age	3 [1,5]	3 [1.25, 5]	3 [1,5]	0.697
(median [IQR])				
Sex(%)				
Male	496 (47.46)	344 (46.36)	152 (50.17)	0.294
Female	549 (52.54)	398 (53.64)	151 (49.83)	
Laterality(%)				
Left	530 (50.72)	380 (51.21)	150 (49.50)	0.665
Right	515 (49.28)	362 (48.79)	153 (50.50)	
Tumor size	110 [83, 135]	110 [81.25, 135]	110 [89, 136]	0.974
(median [IQR])				
Chemotherapy				
No/Unknown	90 (8.61)	60 (8.09)	30 (9.90)	0.408
Yes	955 (91.39)	682 (91.91)	273 (90.10)	
LND	0.00	0.00	0.00	0.667
(median [IQR])	[0.00, 0.00]	[0.00, 0.00]	[0.00, 0.00]	
Regional nodes	5 [3,9]	5 [3,9]	5 [2,9]	0.269
examined (median [IQR])				
Regional nodes	0.00	0.00	0.00	0.652
positive (median [IQR])	[0.00, 0.00]	[0.00, 0.00]	[0.00, 0.00]	
Stage(%)				
Localized	435 (41.63)	308 (41.51)	127 (41.91)	0.578
Regional	379 (36.27)	264 (35.58)	115 (37.95)	
Distant	231 (22.11)	170 (22.91)	61 (20.13)	
Metastasis(%)				
No	831 (79.52)	587 (79.11)	244 (80.53)	0.666
Yes	214 (20.48)	155 (20.89)	59 (19.47)	

*IQR, inter-quartile range*.

We collected a total of 112 children with WT in our hospital. The median age of all children was 3 years old(quartile, 1–5 years old), and the median follow-up time was 22 months (quartile, 12 to 42.5 months). Among them, most patients had undergone chemotherapy (99.1%), the number of positive LNs accounted for 18.8%, and the median number of LNs examined was 2 (quartile, 0–5). The SEER stage of patients was as follows: the localized accounted for 60.71%, the region accounts for 20.54%, and the foreign accounts for 18.75%. The clinicopathological characteristics of the SEER cohort and the CHCMU cohort are shown in Table 2.

**Table 2:** Clinicopathological characteristics of children with WT in SEER cohort and CHCMU.

	**Total**	**SEER cohort**	**CHCMU cohort**	
	***N* = 1157**	***N* = 1045**	***N* = 112**	***p***
Age	3 [1,5]	3 [1,5]	2 [1,4]	0.003
(median [IQR])				
Sex(%)				
Male	553 (47.80)	496 (47.46)	57 (50.89)	0.554
Female	604 (52.20)	549 (52.54)	55 (49.11)	
Laterality(%)				
Left	589 (50.91)	530 (50.72)	59 (52.68)	0.767
Right	568 (49.09)	515 (49.28)	53 (47.32)	
Tumor size	110.00	110.00	120.00	0.251
(median [IQR])	[83.00, 138.00]	[83.00, 135.00]	[87.50, 150.00]	
Chemotherapy(%)				
No/Unknown	158 (13.66)	90 (8.61)	1 (0.89)	<0.001
Yes	999 (86.34)	955 (91.39)	(99.11)	
LND	0.00	0.00	0.00	0.9
(median [IQR])	[0.00, 0.00]	[0.00, 0.00]	[0.00, 0.00]	
Regional nodes	5.00	5.00	2.00	<0.001
examined (median [IQR])	[2.00, 9.00]	[3.00, 9.00]	[0.00, 5.00]	
Regional nodes	0.00	0.00	0.00	0.909
positive (median [IQR])	[0.00, 0.00]	[0.00, 0.00]	[0.00, 0.00]	
Stage(%)				
Localized	503 (43.47)	435 (41.63)	68 (60.71)	<0.001
Regional	402 (34.75)	379 (36.27)	23 (20.54)	
Distant	252 (21.78)	231 (22.11)	21 (18.75)	
Metastasis(%)				
No	927 (80.12)	831 (79.52)	96 (85.71)	0.151
Yes	230 (19.88)	214 (20.48)	16 (14.29)	
Survival months	57 [25, 104]	65 [28, 111]	22 [12, 42.5]	<0.001
(median [IQR])				

*IQR, inter-quartile range*.

### Univariate and Multivariate Cox Regression Analysis

We performed univariate and multivariate Cox regression analysis on patients in the SEER database and revealed age (HR 1.119, 95%CI: 1.039–1.206), LND (HR 4.465, 95%CI: 1.99–10.017), and stage (HR 1.673, 95%CI: 0.661–4.239; HR 3.603, 95%CI: 1.455–8.923) were independent risk factors for the prognosis of children with WT (Table 3). However, we performed univariate and multivariate Cox regression analysis on the patients collected in our hospital, suggesting that positive LNs (HR 2.958, 95%CI: 1.803–4.851), stage (HR 1.731, 95%CI: 0.359–8.345; HR 15.151, 95%CI: 4.744–48.39;) were independent risk factors for prognosis of children with WT (Table 4).

**Table 3:** Univariate and multivariate analyses of CSS in SEER cohort.

	**Univariate**			**Multivariate**		
	**HR**	**95%CI**	***P***	**HR**	**95%CI**	***P***
Age	1.15	1.08–1.23	<0.0001	1.12	1.04–1.21	0.003
Sex						
Male						
Female	0.79	0.44–1.42	0.437			
Laterality						
Left						
Right	0.50	0.27–0.93	0.028			
Tumor size	1.00	1–1.01	0.373			
Chemotherapy						
No/Unknown						
Yes	0.99	0.35–2.75	0.980			
LND	8.31	3.96–17.42	<0.001	4.47	1.99–10.02	<0.001
Regional nodes	0.99	0.95–1.04	0.821			
examined						
Regional nodes	1.27	1.15–1.4	<0.001			
positive						
Stage						
Localized						
Regional	2.57	1.05–6.30	0.039	1.67	0.66–4.24	0.278
Distant	6.50	2.79–15.14	<0.001	3.60	1.46–8.92	0.006

**Table 4:** Univariate and multivariate analyses of CSS in CHCMU cohort.

	**Univariate**			**Multivariate**		
	**HR**	**95%CI**	***P***	**HR**	**95%CI**	***P***
Age	1.12	0.95–1.33	0.175			
Sex						
Male						
Female	0.88	0.36–2.17	0.778			
Laterality						
Left						
Right	1.10	0.44–2.71	0.843			
Tumor size	1.00	0.99–1.01	0.740			
LND	3.42	0.98–11.92	0.054			
Regional nodes	1.07	0.98–1.16	0.144			
examined						
Regional nodes	2.36	1.65–3.37	<0.001	2.96	1.80–4.85	<0.001
positive						
Stage						
Localized						
Regional	2.16	0.52–9.04	0.292	1.73	0.36–8.35	0.494
Distant	11.05	3.79–32.26	<0.001	15.15	4.74–48.39	<0.001

### Nomogram Construction and Validation

**Figure 2:**
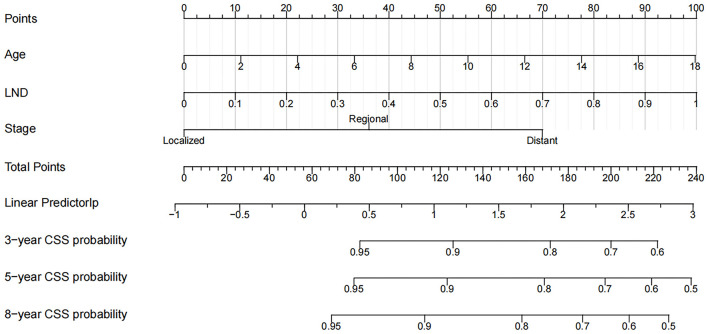
Nomogram for 3-, 5-, and 8-year CSS of children with WT.

**Figure 3:**
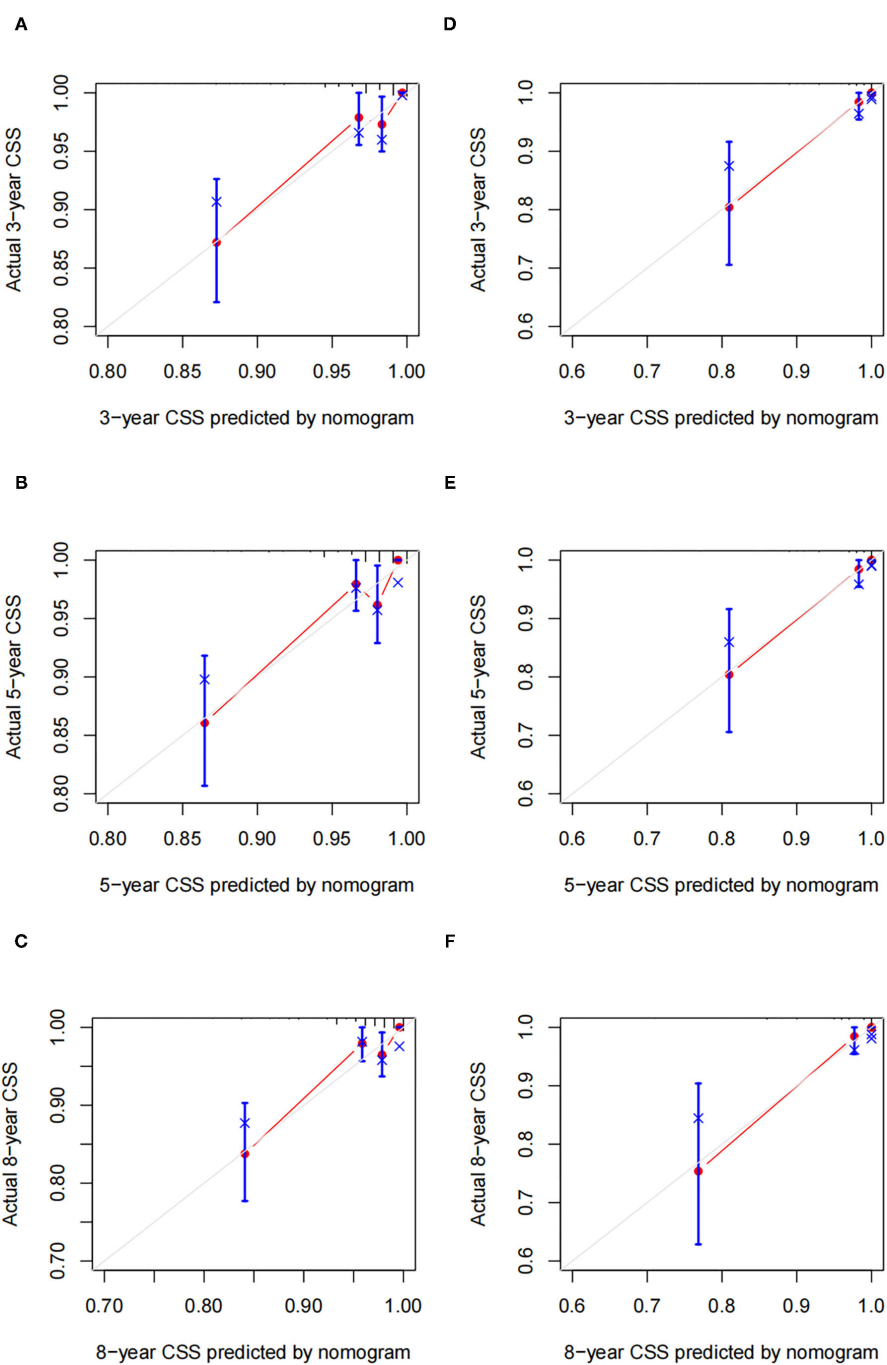
Calibration plots of a nomogram for 3-, 5-, and 8-year CSS in training cohort **(A–C)** and validation cohort **(D–F)**.

**Figure 4:**
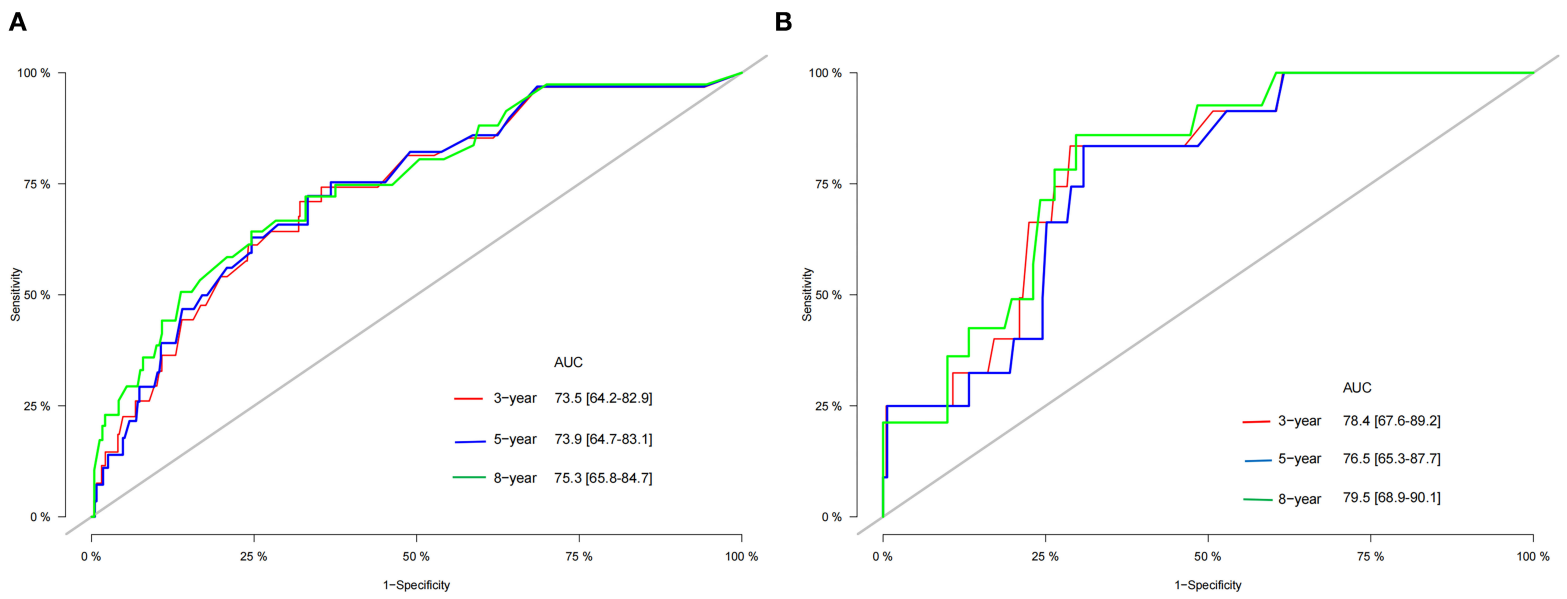
The AUC of 3-, 5-, and 8-year in the training cohort **(A)** and validation cohort **(B)**.

**Figure 5:**
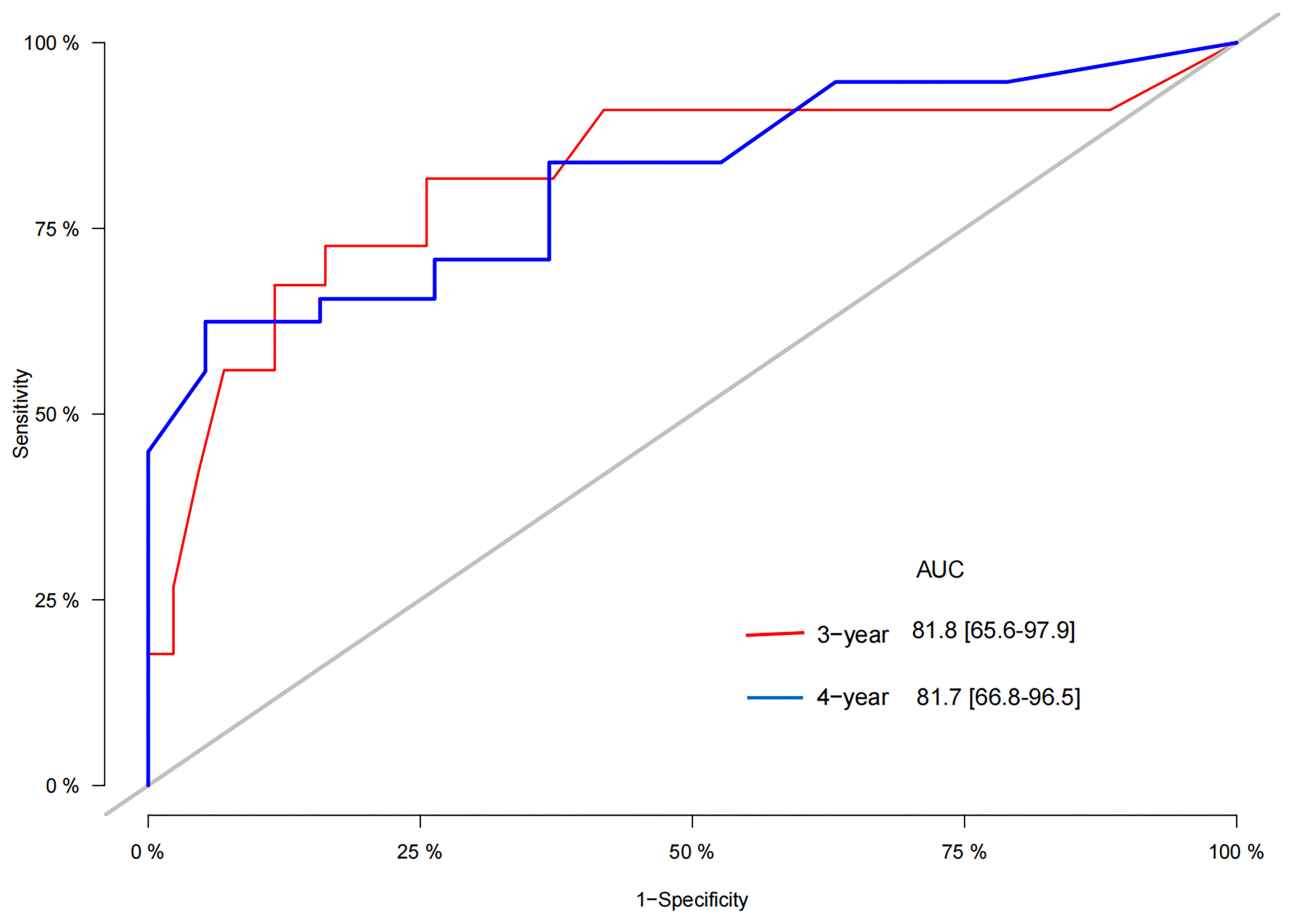
The AUC of 3-and 4-year of children in the CHCMU.

We used the independent risk factors identified by patients in the SEER database to construct a nomogram (Figure 2). The nomogram indicated that age and LND were the most significant risk factors affecting patient survival, followed by stage. The calibration curves of the training set and the validation set indicated that the predicted value was consistent with the observed value, which suggested that the nomogram has good accuracy (Figures 3A–F). The C-index of the training set and the validation set were 0.811 (95% CI, 0.738–0.884) and 0.937 (95% CI, 0.884–0.990), respectively. The C-index of the external validation set was 0.902 (95% CI, 0.837–0.967). The AUC of the training set, validation set (Figures 4A,B) and external validation (Figure 5) set showed similar results, suggesting that the nomogram has good accuracy and discrimination. The calibration curves of the training set and the validation set indicated that the predicted value was consistent with the observed value, which suggested that the nomogram has good accuracy.

### Clinical Application of the Nomogram

**Figure 6:**
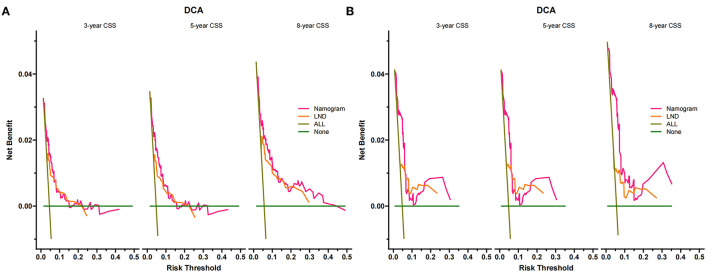
Decision curves of the nomogram predicting CSS in the training cohort **(A)** and the validation cohort **(B)**. The x-axis is the threshold probability, and the y-axis is the net income. The green line indicates that no patients have died, and the dark green line indicates that all patients have been killed. When the threshold probability is between 5% and 40%, the net benefit of the model exceeds all deaths or no deaths.

**Figure 7:**
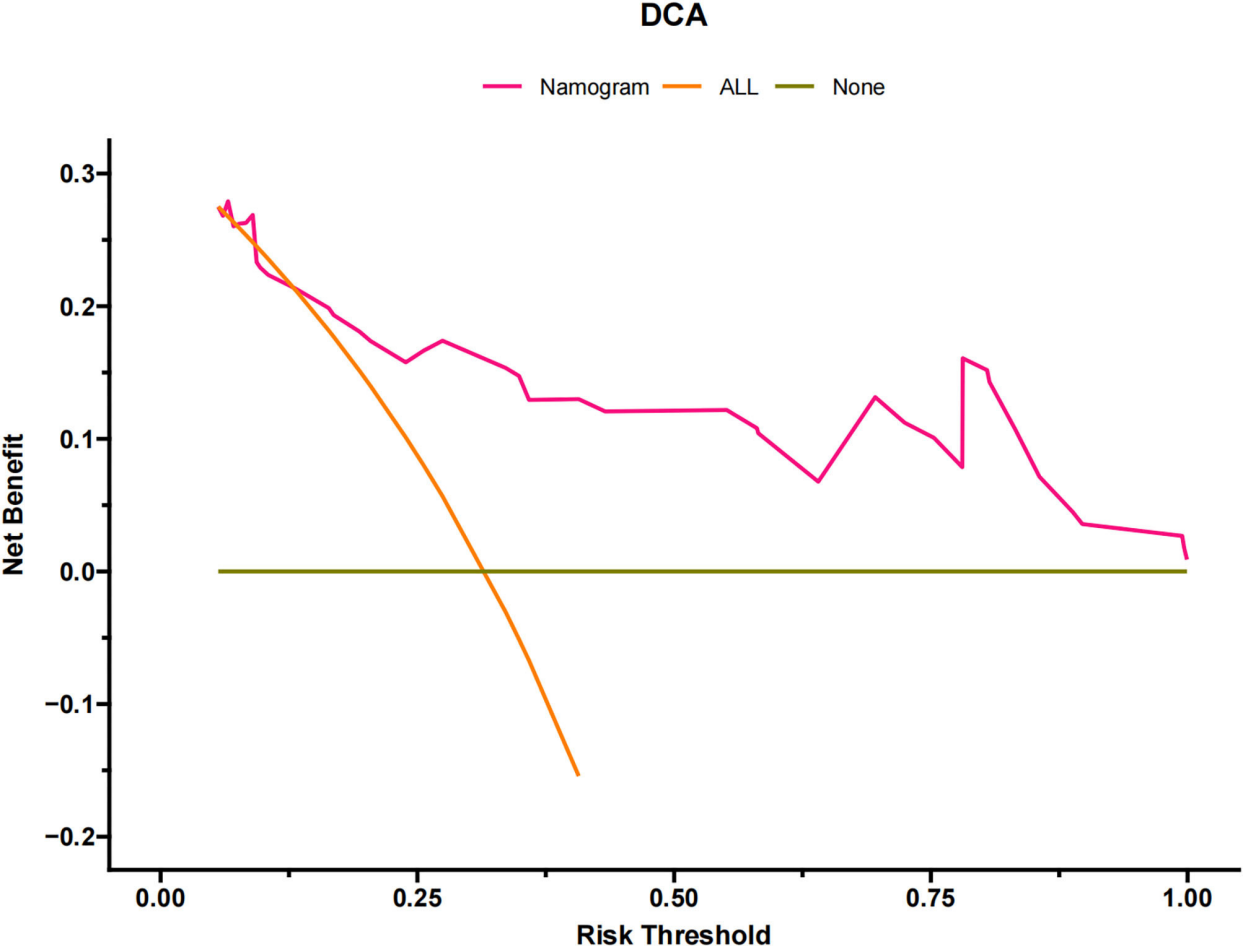
Decision curves of the nomogram predicting CSS in CHCMU.

**Figure 8:**
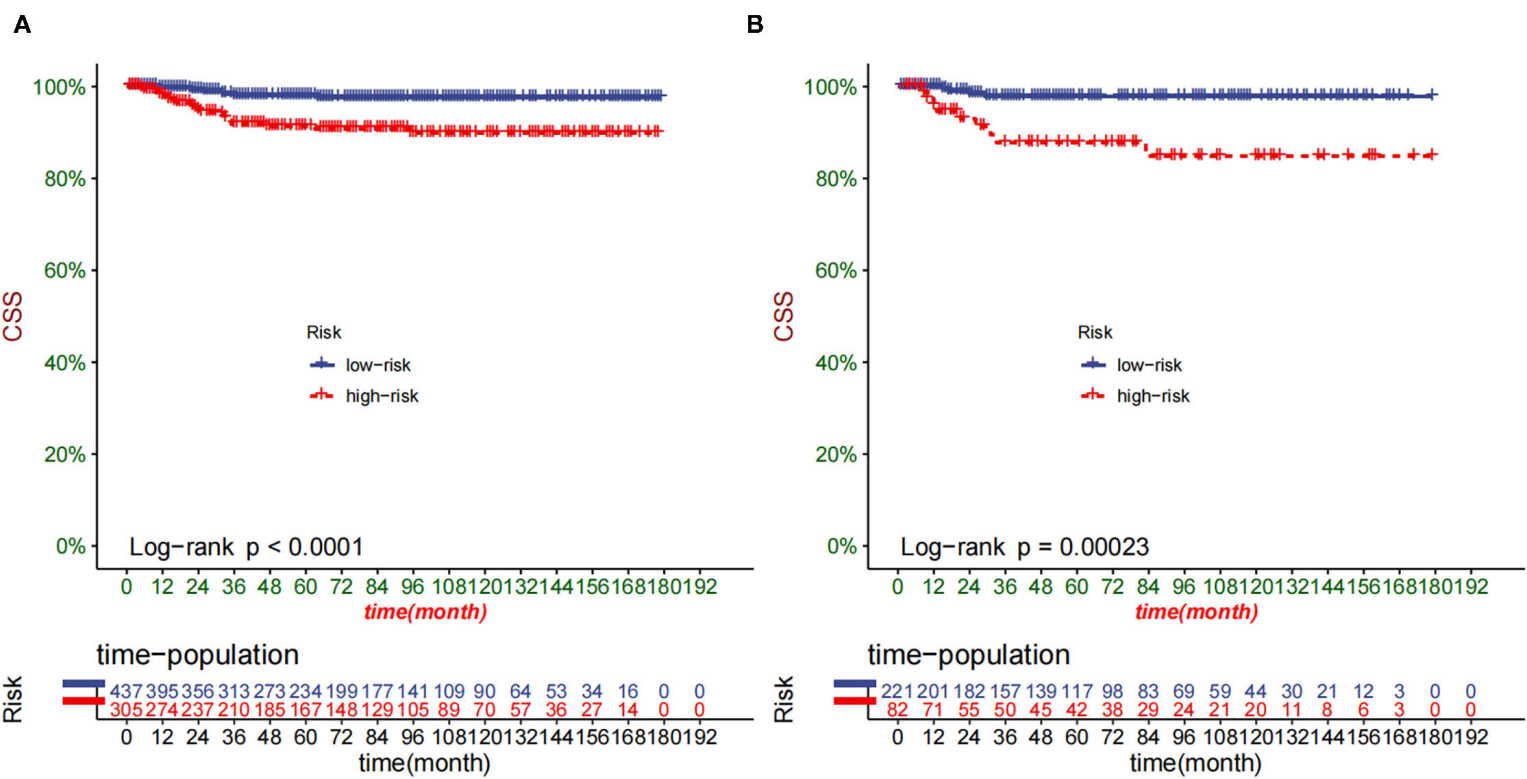
Kaplan–Meier curves of CSS for children in the low- and high-risk groups in the training set **(A)** and validation set **(B)**.

**Figure 9:**
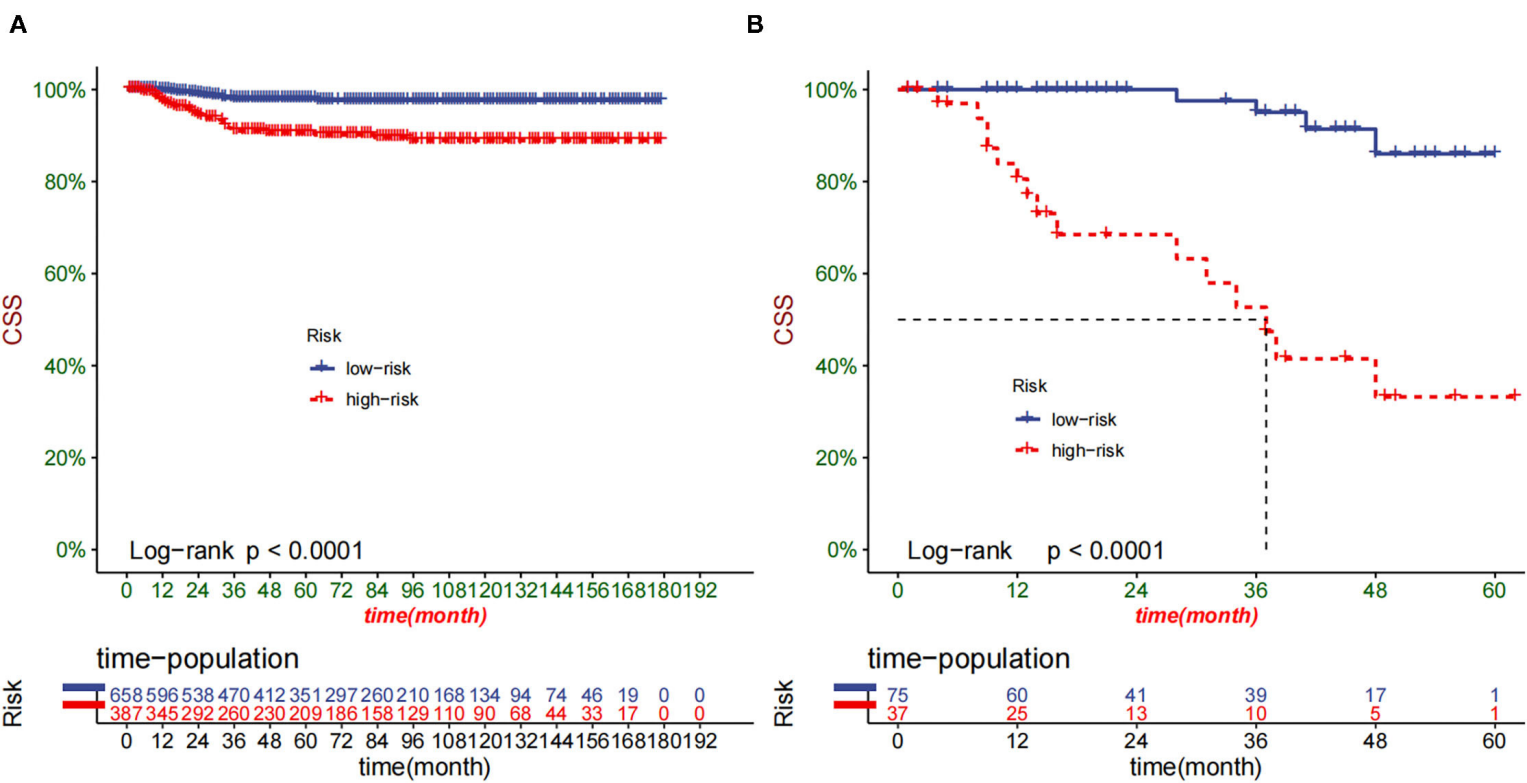
Kaplan–Meier curves of CSS for children in the low- and high-risk groups in SEER **(A)** and CHCMU cohort **(B)**.

**Figure 10:**
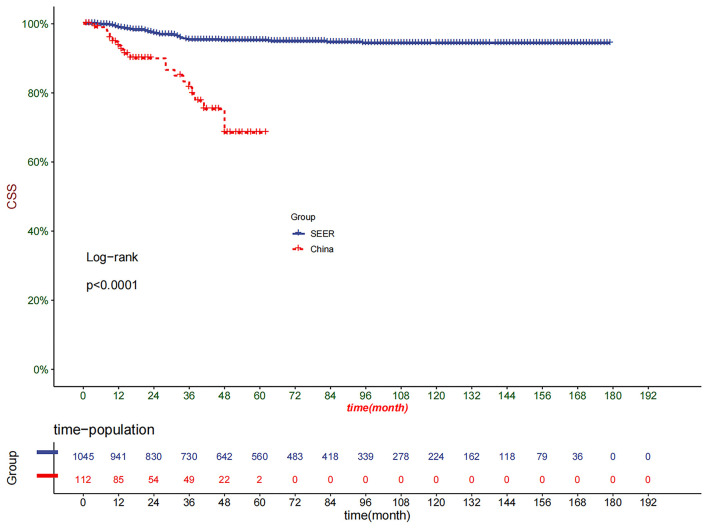
Kaplan–Meier curves of CSS for children in SEER cohort and CHCMU cohort.

The DCA of the training set and the validation set indicated that the nomogram had an excellent clinical value (Figures 6A,B), and the DCA of the external validation set also proved the potential clinical value of the model (Figure 7). According to the risk value in the nomogram, we divided the patients into low-risk (≤60.2) and high-risk (>60.2) groups. The Kaplan-Meier curve and log-rank test showed that the high-risk group had a lower survival rate in the training and validation sets (Figures 8A,B). Similarly, the overall cohort and external validation set suggested that the high-risk group had a lower survival rate (Figures 9A,B). It proved that the nomogram had good discrimination. In addition, the Kaplan-Meier curve suggested that the survival rate of children in CHCMU was significantly lower than that of children in the SEER database (Figure 10).

## Discussion

For childhood kidney tumors, COG-RTC and SIOP-RTSG are the two most authoritative academic organizations for clinical research. Although the two adopted different treatment strategies for children with WT (surgery first vs. chemotherapy first), the therapeutic effects obtained were very close, and the long-term survival rate of patients was over 90% (23, 24). However, the total treatment capacity of Chinese children with WT is still insufficient, and only in some children's cancer centers, the 5-year survival rate of children reach 80% (25, 26). Although multidisciplinary combined treatment has dramatically increased the survival rate of children with WT, some high-risk children have low survival rates, especially in developing countries. Studies had shown that tumor rupture, no lymph node examined, insufficient chemotherapy intensity, insensitivity to chemotherapy were high-risk factors for the prognosis (27, 28). At present, although there was a nomogram to predict the CSS of patients with WT (10), there was no special nomogram for the survival prognosis of WT in children. Therefore, we used SEER database patients to screen independent risk factors and established a nomogram to predict the prognosis in children.

This study found that age was an independent risk factor for WT in children, and older age meant higher mortality, similar to previous studies (29). In addition, LND was also an independent risk factor in the study, and the previous research had also proved that LND was an important factor (30). SEER stage was also a risk factor, and patients with distant metastases had a worse prognosis. According to the stratification of risk value, the Kaplan-Meier curve and log-rank test proved that the nomogram had an excellent discriminating ability. Multivariate Cox regression analysis suggested that the risk factors of children in our hospital were significantly different from those in the SEER database. Race may be a potential factor, even though the race had never been a risk factor in previous studies (30, 31). In addition, our follow-up time was significantly shorter, which was also one of the reasons for the deviation of the results.

In the cases in our hospital, we found that the children were younger than patients in the SEER database, which may be related to the high incidence of WT in China (8). In addition, the number of LNs examined in our hospital was lower than that in the SEER database. The children in our hospital were young, and the lymph nodes were challenging to identify during the operation, resulting in a significant decrease in the number of LNs examined. Recent studies have shown that failure to sample lymph nodes during tumor resection was associated with increased tumor recurrence rates (31, 32). Due to the occult lymph node metastasis in some low-stage WT, these children have not been appropriately staged, resulting in insufficient treatment intensity. This was also a reason for the low survival rate of WT in Chinese children.

The construction of a nomogram requires a large number of patients to achieve sure accuracy and reliability. There were only a small number of patients with WT in our hospital, which cannot meet the needs of nomogram construction. The SEER database is a cancer database covering 28% of the US population, with many patients (21). So, the structure of the nomogram was based on patients from the SEER database in the United States. Due to ethnic differences and the baseline differences between the children in our hospital and the SEER database. However, on the contrary, external validation showed that this prediction model still had good accuracy and discrimination. The clinicopathological variables included in this nomogram were easy to obtain and were friendly to both doctors and patients.

Our study still has many limitations and deficiencies. First of all, there are still many variables in the SEER database that are not available, such as 1p and 16q gene mutation factors, which are essential gene variants that affect the prognosis of WT (33). A better model can be constructed if variables with genetic mutations are added. Secondly, because our study is a retrospective study, selection variants are difficult to avoid, such as the lack of tumor size in many patients, which results in a certain degree of bias. However, a previous study suggested that tumor size has a negligible effect on the prognosis (10). Finally, although we conducted external validation, the children's follow-up time was short, making it impossible to validate the long-term prognosis. Long-term prognostic prediction validation and even prospective clinical trials are necessary. With the improvement of medical technology, the prediction model will have some deviation. The prediction model we built needs to be constantly updated in the future to improve accuracy.

## Conclusions

We constructed a nomogram to predict the prognosis of children with WT and We found that age, LND, and stage were independent risk factors for the prediction in children. Internal validation and external validation had been conducted to prove the nomogram has good accuracy and reliability. However, we need to further collect patients to improve the accuracy of the model in the future.

## Data Availability Statement

The original contributions presented in the study are included in the article/supplementary material, further inquiries can be directed to the corresponding author.

## Author Contributions

XT and JW: conceptualization. JW: methodology, investigation, and supervision. JT: software and formal analysis. XT, ML, and ZZ: validation. DH: resources. LJ: data curation. XT: writing—original draft preparation. ML: essay—review and editing. ZZ: visualization. DH: project administration and funding acquisition. All authors have read and agreed to the published version of the manuscript. All authors contributed to the article and approved the submitted version.

## Funding

This research was funded by the Special Key Project of Chongqing Technology Innovation and Application Development (No. Cstc2019jscx-tjsbX0003).

## Conflict of Interest

The authors declare that the research was conducted in the absence of any commercial or financial relationships that could be construed as a potential conflict of interest.

## Publisher's Note

All claims expressed in this article are solely those of the authors and do not necessarily represent those of their affiliated organizations, or those of the publisher, the editors and the reviewers. Any product that may be evaluated in this article, or claim that may be made by its manufacturer, is not guaranteed or endorsed by the publisher.
